# Two ways to overcome the three social dilemmas of indirect reciprocity

**DOI:** 10.1038/s41598-020-73564-5

**Published:** 2020-10-08

**Authors:** Isamu Okada

**Affiliations:** 1 Faculty of Business Administration, Soka University, Tokyo, Japan; 2 Department of Information Systems and Operations, Vienna University of Economics and Business, Wien, Austria

**Keywords:** Human behaviour, Social evolution

## Abstract

Indirect reciprocity is one of the main principles of evolving cooperation in a social dilemma situation. In reciprocity, a positive score is given to cooperative behaviour while a negative score is given to non-cooperative behaviour, and the dilemma is resolved by selectively cooperating only with those with positive scores. However, many studies have shown that non-cooperation with those who have not cooperated also downgrades one's reputation; they have called this situation the scoring dilemma. To address this dilemma, the notion of justified punishments has been considered. The notion of justified punishment allows good individuals who defect against bad co-players to keep their standing. Despite numerous studies on justified punishment, it is unknown whether this solution leads to a new type of dilemma because reputations may be downgraded when the intent of punishment is not correctly communicated. The dilemma of punishment has so far been rarely analysed, and thus, the complete solution of the mechanism for evolving cooperation using the principle of indirect reciprocity has not been found yet. Here, we identify sufficient conditions to overcome each of the three dilemmas including the dilemma of punishment to maintain stable cooperation by using the framework of evolutionary game theory. This condition includes the principle of detecting free riders, which resolves the social dilemma, the principle of justification, which resolves the scoring dilemma, and the principle of generosity, which resolves the dilemma of punishment. A norm that satisfies these principles can stably maintain social cooperation. Our insights may offer a general assessment principle that applies to a wide range of subjects, from individual actions to national decisions.

## Introduction

Humans care about their own reputation and the reputation of others^1^. They often strive to act in a way that would be considered as good by other community members. However, always acting cooperatively may not be possible—or even desirable—for two reasons. One is the loss incurred to oneself by giving alms. Here, cooperation refers to the act of paying a cost to benefit others^2,3^. Traditional game theory says that people who always act cooperatively are dominated by (first-order) free riders who do not cooperate with others. A social dilemma^4–6^ emerges in a situation in which cooperative societies have a higher social welfare than non-cooperative societies while there are economic incentives to betray. The dilemma appears in a variety of situations, from free-riding public transport to restricting people's actions to fight infectious diseases. Moreover, it is not restricted just to human behaviour; for example, it affects the behaviours of ants^7^ and even viruses^8^. Despite the teachings of game theory, the reality is that living things behave cooperatively in many social dilemma situations, and this mystery has been discussed by researchers in many disciplines and has been the topic of numerous studies on the evolution of cooperation^9–12^.

The findings of the studies on the evolution of cooperation reveal a second reason why it is not always advisable to behave cooperatively. That is, it benefits free riders. Many studies explored the conditions under which cooperation is evolutionarily stable. However, a number of theoretical and experimental studies have found that free riders can also gain if there are perfect cooperators who cooperate unconditionally, allowing free riders to invade cooperative regimes^13–18^. For this reason, researchers call perfect cooperators second-order free riders and are interested in eliminating them. They think that not taking action to punish free riders is the same as free riding on public goods. They propose that discriminating between free riders and cooperators and punishing the former is the key to maintaining stable cooperation. Whether it is in personal^19,20^ or institutional^21–23^ punishment systems, if punishment is essential to maintaining cooperation, the group (at least part of the group) must punish. Thus, we return to the first question. If punishment is essential while caring about the reputations of others, how should punishment be assessed?

Indirect reciprocity^13,24–31^ is an evolutionary mechanism of cooperation that expresses the idea of using people's reputation to identify those who should act cooperatively. The idea is simply to give people two reputation labels, good and bad, and try to maintain cooperative regimes by cooperating only with those with good reputation labels. The heavy use of reputation systems in personal online sales is empirical evidence of the prevalence of this mechanism. Usually, the assessment rules of this reputation system are simple: Give good credit to those who do a good deal and bad credit for misconduct. Such a simple rule is called image-scoring and has been considered in both theoretical analyses and experiments^25,26,32,33^.

Despite its simplicity and apparent effectiveness, image-scoring has three problems^25,34^ as follows. One is that the image-scoring rule adopters are indistinguishable from perfect cooperators if the population consists of these two types, and the adopters allow for invasion of the perfect cooperators. Second, in obeying image-scoring rules, people must as disciplinary actions punish bad people who behave uncooperatively, but the practice of doing so may also downgrade their own reputation under image-scoring rules. Third, unintentional defection may occur due to either accident or lack of resources even if they may want to cooperate. As a result, a chain of non-cooperation occurs, and the degree of social cooperation continuously decreases. The combination of these three problems causes the image-scoring rule to lose its evolutionary stability, as it allows a society to be invaded by second-order free riders (perfect cooperators) and subsequently by first-order free riders (perfect defectors). This Achilles' heel of image-scoring^35–37^ is called the scoring dilemma^26–28,34,35,38^, and many studies have been devoted to it.

Resolving the scoring dilemma requires an assessment taking into account the intent of non-cooperation. Non-cooperation is justified if it is possible to identify whether it is simply malicious or a punishment for the recipient's bad reputation. To theoretically analyse the idea of justified punishment^27,34,39–43^, theorists use an assessment function to determine reputations. While the domain of the function in image-scoring is actions only, the domain has been extended to include reputation information of the recipient in order to consider justified punishment. These analyses have made it clear that a rule which assesses justified non-cooperation as good has evolutionary stability. In particular, Otsuki and Iwasa^39,44^ identified successful norms, called the leading eight, by exhaustively analysing these rules. These norms have in common the principle that non-cooperative behaviour against bad recipients is assessed as good.

However, almost all of the previous studies make public assessment assumptions, and the reputations of recipients are known to all^39,42,44–50^. In reality, this is an overly unnatural assumption, overlooking important considerations in regard to justification. That is, punishment is justified only when both the doer and the observer agree on the recipient's reputation. If the observer rates the recipient as good while the doer assesses the recipient as bad, the intention of punishment is not communicated to the observer. Non-cooperation is perceived as a malicious action toward the good, and the doer loses its reputation even if the norm employs the principle of justified punishment. In other words, the punishment creates a dilemma. This dilemma of punishment refers to the loss of reputation when the intention is not correctly transmitted.

**Figure 1:**
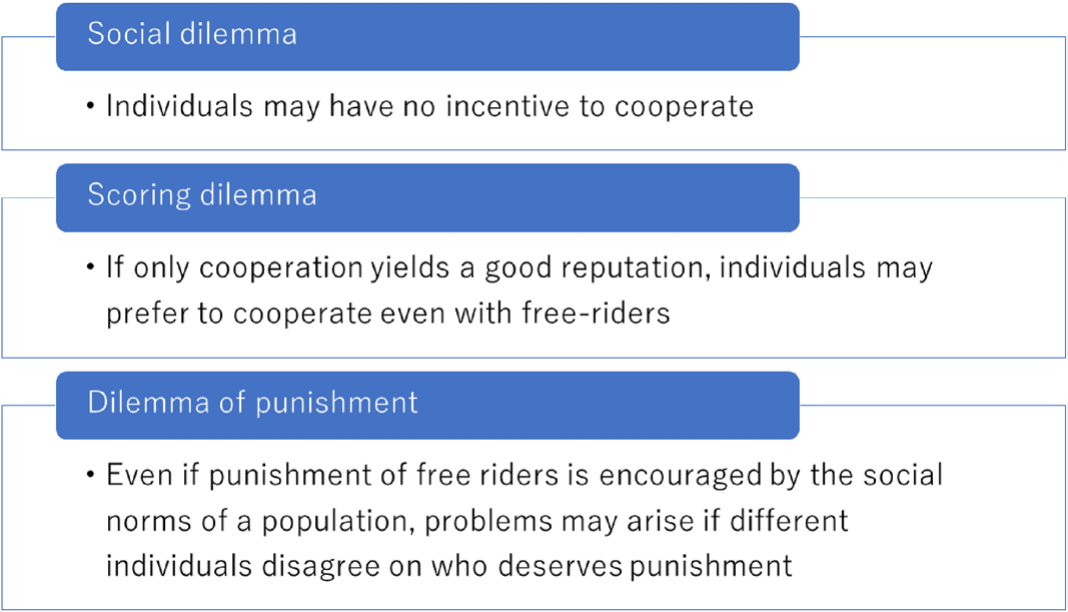
Conceptual explanation of the three dilemmas: social, scoring, and punishment.

In summary, when trying to resolve any social dilemma in the framework of indirect reciprocity, it is necessary to overcome the dilemma of punishment as well as the scoring dilemma. However, the norms that resolve all three dilemmas (Fig. 1) have attracted little academic interest because of indifference to the dilemma of punishment, and no one has identified the conditions that resolve those three dilemmas. Here, we show the conditions resolving the dilemma of punishment by using an indirect reciprocity model in the framework of evolutionary game theory. The previous analyses of indirect reciprocity models have used a public assessment scheme which does not capture the dilemma of punishment. Using our model, we analyse this dilemma in the framework of a private assessment scheme, which is a relaxation of the public one. The results show that the principle of generously assessing cooperative behaviour of badly reputed recipients overcomes the dilemma of punishment. The finding is paradoxical, in that the elimination of the dilemma of punishment should be restricted not only in terms of punishment but also in assessing cooperative behaviour. Moreover, we discover another way to resolve the scoring dilemma using a private assessment scheme, while the previous studies show that the principle of justified punishment is needed to resolve the scoring dilemma. We identify the characteristics of norms that can resolve all three dilemmas for maintaining stable cooperation.

## Model description and analysis

We sought to determine which assessment functions are able to resolve social dilemmas and maintain stable cooperation. An assessment function is a function that determines a reputation label when a certain action is performed on a recipient having a certain reputation label. In order to perform the simplest analysis, the domain of the assessment function is limited to the minimum, and only deterministic functions are handled. As described in the Methods Section, a total of 256 types of assessment function are defined.

Let us outline the assessment function analysis (a detailed description is given in the Methods Section). For each assessment function, we model a population of infinite players without an interaction structure. In each model, there are three types of player: unconditional cooperators (ALLC), unconditional non-cooperators (ALLD) and discriminators (DISC). In a social dilemma situation, ALLD is called a first-order free rider because it never cooperates. ALLC does not punish and is therefore called a second-order free rider that allows ALLD to invade. We find stable cooperative regimes by analysing the replicator dynamics of the three types of player, maximizing their expected payoffs. In this situation, there is a certain fraction of DISCs and their cooperation rate remains high in a steady state (a state where the population ratio of each type is constant or periodic in time).

As mentioned above, our analysis considers a private assessment scheme, as the dilemma of punishment occurs in situations where reputation labels are not shared. In this scheme, there are players who can observe a game and players who cannot. If players observe a game, they update the reputation of the doers of the game and do not communicate the updated reputation to other players. Therefore, reputation labels are updated privately. To avoid confusion, we will refer reputation labels as private labels. Assuming an infinite population, this scheme requires the solution of an infinite number of equations, so solitary observations are assumed for deriving rigorous solutions without approximation^38,49,51^. This assumption limits the number of players that can observe a game to a finite number.

While a rigorous analysis of all assessment functions is performed in the Methods Section and in the Supplementary Information, here we introduce parameters and notation to help readers understand our results. We use two game parameters: cost of cooperation (*c*) and benefit to its recipients (*b*). Moreover, we have two types of error, errors in implementation (*e*_*1*_) and errors in assessment (*e*_*2*_). In the following figures, an assessment rule is denoted as a four-letter string (For example, *[GBGB]*). The meanings of each letter are as follows. The first letter is a response to a situation in which the donor chooses to give *C* to someone with a *G* label. The second letter is a response to the case in which the donor chooses to give *D* to someone with a *G* label. The third letter is a response to the case in which the donor chooses to give *C* to someone with a *B* label, and the fourth letter is a response to the case in which the donor chooses to give *D* to someone with a *B* label. The set of responses is denoted as *U* = *{G,B,K,R}*, the symbols of which indicate 'Assign *G*' , 'Assign *B*', 'Keep the label unchanged', and ‘Reverse the label between *G* and *B*’, respectively. While DISC players use a specific assessment rule, their action rules are simply assumed to be cooperate with good individuals and to defect against bad individuals.

**Figure 2:**
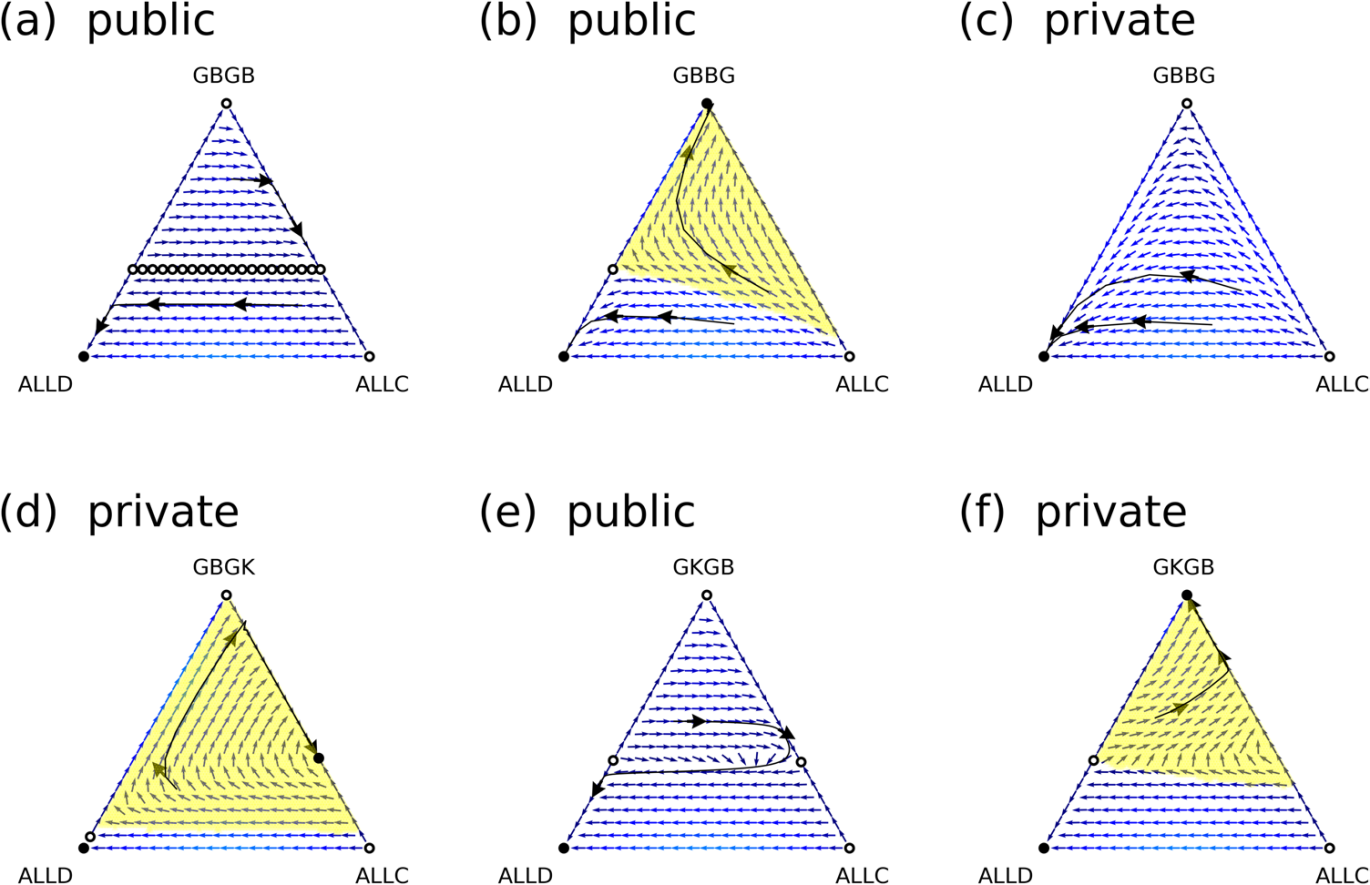
Analytic results of representative norms. Each triangle represents a simplex of the state space, where *x*, *y*, and *z* are non-negative real numbers denoting the frequencies of ALLC, ALLD, and DISC, respectively. The arrows in the triangles show the direction of replicator dynamics at each point. Trajectories following the dynamics are also drawn. If there is a cooperative stable point, the basin of attraction is shown in yellow. Circles denote rest points. Filled circles correspond to stable rest points. Panels (**a**), (**b**), and (**e**) are the results of the public assessment scheme, and panels (**c**), (**d**), and (**f**) are the results of the private assessment scheme. The assessment functions for each panel are (**a**) *[GBGB]*, (**b**) and (**c**) *[GBBG]*, (**d**) *[GBGK]*, and (**e**) and (**f**) *[GKGB]*. The parameter values are *b* = *3*, *c* = *1*, *e*_*1*_ = *1%*, and *e*_*2*_ = *1%*. This image is made by Python 3.

**Figure 3:**
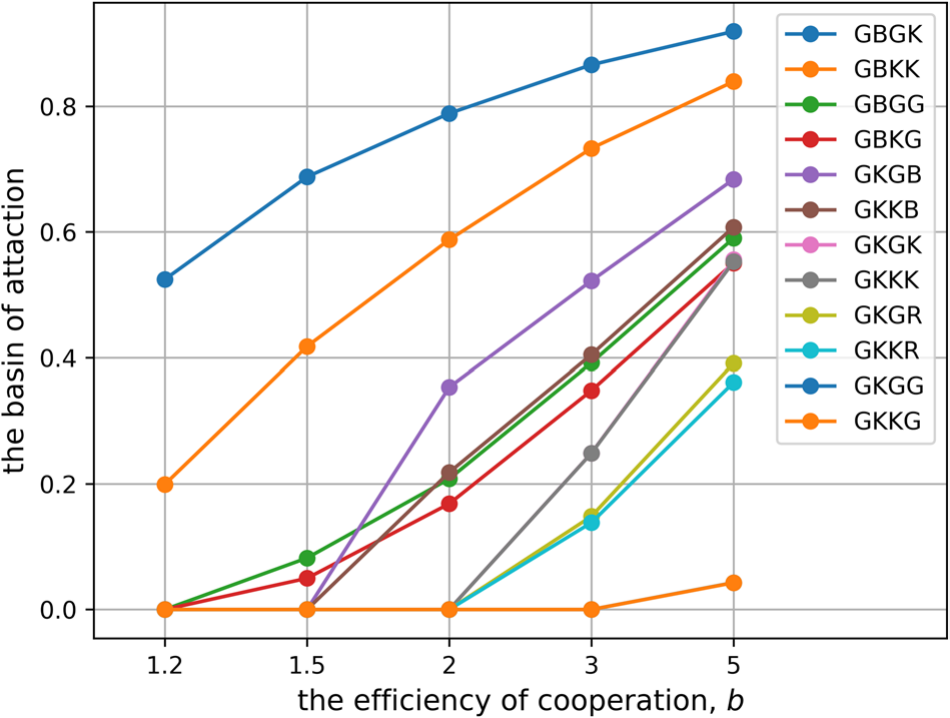
Stably cooperative norms depend on the efficiency of cooperation. The horizontal axis represents the values of *b*, while the vertical axis represents the area of the basin of the attraction if there is a cooperative stable point. The parameter values are *c* = *1*, *e*_*1*_ = *1%*, and *e*_*2*_ = *1%*. This image is made by Python 3.

Figure 2 shows results for representative assessment functions. Panel (a) in the figure shows that the scoring dilemma emerges, although resolving the social dilemma requires the detection of free riders by scoring. Panel (b) shows a solution of the scoring dilemma by justified punishment. However, this solution can only be executed in public assessment schemes. Panel (c) shows that the same evaluation function as in panel (b) results in the dilemma of punishment and collapse of the cooperative regime in a private assessment scheme. Panel (d) shows that a norm adopting the principle of generosity resolves the dilemma of punishment and maintains stable cooperation. As a second way of resolving the scoring dilemma, we show the results of another justified punishment focusing on the private labels of doers. If a public assessment scheme is assumed, such a justification resolves neither the scoring dilemma nor the dilemma of punishment as shown in panel (e). If a private scheme is assumed, however, it resolves both dilemmas and maintains stable cooperation, as shown in panel (f). Moreover, Fig. 3 shows that the norms that maintain stable cooperation depend on the efficiency of cooperation.

## The first way to resolve the three dilemmas

There are only two assessment functions that can stably maintain cooperation even under the condition of lowest cooperative efficiency (Case of *b* = *1.2* in Fig. 3). On the basis of the features of these functions, we find norms with the following four conditions as the first solution to resolve the three dilemmas.

(1) Assign a good private label to someone who helped a good person.

(2) Assign a bad private label to someone who defected against a good person.

(3) Assign a good private label to someone who helped a bad person (or leave the respective private label unchanged).

(4) Assign a good private label to someone who defected against a bad person.

To sum up, it can be said that an assessment rule that rigorously evaluates the actions of reputable recipients and generously evaluates the actions of disreputable ones can maintain stable cooperative regimes.

Conditions (1) and (2) are consistent with the results already clarified by previous analyses of public assessment schemes. As shown in panels (a) and (b) of Fig. 2, the image-scoring rule and the rule most commonly used by the leading eight norms also satisfy these conditions. These conditions resolve the social dilemma by detecting free riders and is a fundamental principle for maintaining a stably cooperative regime using the mechanism of indirect reciprocity. Conditions (2) and (4) show that the scoring dilemma is resolved by justified punishment because these conditions express the principle of justified punishment that non-cooperative behaviour towards those who have a good reputation is not recognized as a punishment, while non-cooperative behaviour towards those who have a bad reputation is recognized. In addition, condition (4) is more restrictive than the results of the previous analyses of public assessment schemes.

The conditions of norms for maintaining stable cooperation are relaxed as cooperation becomes more efficient. When cooperation efficiency (*b*) reaches *1.5*, four norms maintain stable cooperation. Accordingly, condition (4) is relaxed to (4′).

(4′) Assign a good reputation to someone who defected against a bad person (or leave the respective private label unchanged).

Condition (4′) is consistent with the condition identified in the exhaustive analysis of the public assessment scheme.

This study reveals that condition (3) can resolve the dilemma of punishment and maintain a stable cooperative regime. The point of the dilemma is to downgrade the reputations of punishers. Because conditions (1), (2), and (4) are already restricted to resolving the first two dilemmas, the use of the freehand condition (3) in an assessment function is required to raise the reputations of punishers who are downgraded by the dilemma of punishment.

However, condition (3) opens the door to invasion by ALLC. A population consisting of DISC is susceptible to being invaded by ALLC due to the decline in their payoffs of downgrading private labels. In this situation, if a further positive evaluation of cooperative behaviour is made in accordance with condition (3), ALLCs will invade when there are many assessment functions. On the other hand, DISCs can invade a population consisting of ALLCs. This is because unintended non-cooperation or misperception of the reputation that occurs with a small probability makes DISCs slightly less cooperative and can reduce the cost of cooperation.

In other words, the principle of generous assessment for resolving the dilemma of punishment raises another problem: the invasion of second-order free riders (ALLCs). However, the co-existence of ALLCs and DISCs does not create a social dilemma if the first-order free riders (ALLD) cannot invade the population. Enough DISCs in a situation of co-existence to discriminate against non-cooperation of ALLDs can prevent the social dilemma (that is, invasion by ALLDs) caused by resolving the dilemma of punishment. As a result, such norms completely resolve all problems arising from the social dilemma.

## The second way to resolve the three dilemmas

If the efficiency of cooperation increases as shown in the case of *b* >  = *2* in Fig. 3, other assessment functions can be used to maintain stable cooperation. This result shows another way to resolve the scoring dilemma. In the new evaluation function, condition (2) is changed to (2′), while conditions (1) and (3) are maintained. Moreover, condition (4) is not required; i.e., any response is fine to someone who defected against a bad person.

(2′) Leave the respective private label unchanged on someone who defected against a good person.

Condition (2′) substitutes for conditions (2) and (4). In conditions (2) and (4), justified punishment is confirmed according to the recipient's private label, whereas in (2′), punishment is confirmed according to the reputation information of the doer. That is, in the second way, a label remains good if the non-cooperator has a good private label, while the punishment function is activated if the non-cooperator has a bad label.

However, condition (2′) alone cannot completely resolve the scoring dilemma, as shown in panel (e) of Fig. 2. Just paying attention to the reputation information of the doer cannot completely identify the justification. However, if condition (3) is given in the private assessment scheme, both the scoring dilemma and the dilemma of punishment are simultaneously resolved. This is because condition (3) provides an advantage to the invading ALLCs, whereas elimination of (4) prevents a DISC's private labels from being downgraded. In other words, the second way resolves the three dilemmas, including the dilemma of punishment, without an invasion of ALLCs (or an invasion of ALLDs facilitated by ALLC) by adopting the principle of generous assessment.

**Figure 4:**
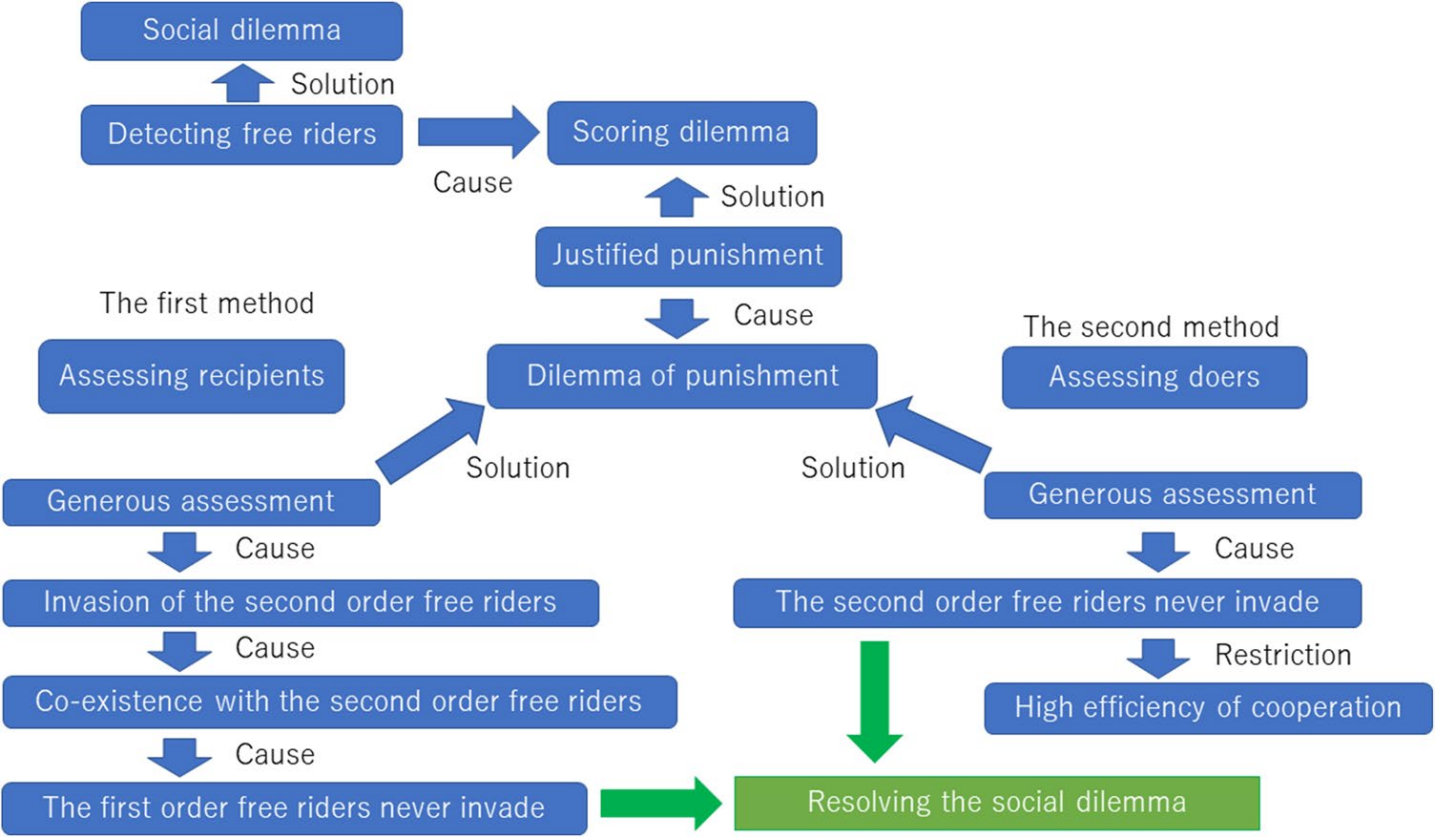
Diagram of mechanisms of resolving the social dilemma, scoring dilemma, and dilemma of punishment.

In summary, conditions (2′) and (3) mean that the scoring dilemma and the dilemma of punishment are resolved at the same time, and that this is the second solution that does not generate a further social dilemma. However, this solution has two limitations compared to the first. One is that it cannot work unless the efficiency of cooperation is high. The other is the basin of attraction which maintains stable cooperation on the population composition ratios (an area coloured in yellow of Fig. 2) is smaller than the norms following the first solution. Figure 4 summarizes these two solutions.

## Discussion

When resolving social dilemmas with indirect reciprocity, two other dilemma types emerge, and consequently, complete resolution of the social dilemma requires the resolution of all three. In this study, we explored the conditions for resolving the dilemma of punishment using the simplest model and found that it can be resolved by imposing a constraint where cooperative behaviour towards recipients with a bad reputation is assessed as good. We thus completely resolved the social dilemma and found the necessary conditions of assessment rules for maintaining stable cooperation.

As shown in Fig. 4, when the social dilemma is resolved using the mechanism of indirect reciprocity, a norm satisfying the principle of detecting free riders is required. However, this principle leads to another issue called the scoring dilemma. We found two methods for resolving this dilemma.

The first method is a solution using a norm which considers to whom a punishment is given, in other words, the principle of justified punishment considering recipients. This solution, however, raises a new issue called the dilemma of punishment. Resolving the dilemma of punishment requires the additional principle of generous assessment wherein donors who are cooperative to those with a bad reputation are generously assessed. Although this principle resolves the dilemma of punishment, it does not prevent invasion by second-order free riders. However, to facilitate co-existence of the second-order free riders and norm-adopters, there is a norm that can activate the punishment function so that first-order free riders cannot invade the population. This makes it possible to completely resolve the social dilemma.

We showed that a certain percentage of unconditional cooperators are required to completely resolve the social dilemma in the first method. In the studies so far, such unconditional cooperators have been excluded because they are second-order free riders. If an evaluator cannot share reputation labels with others, such a situation leads to the dilemma of punishment. In the situation, the norms alone cannot achieve stability, but the co-existence of second-order free riders (unconditional cooperators) can maintain stable cooperation and at the same time prevent invasion by first-order free riders. This means that the norm-adopters and second-order free riders play different roles in maintaining stable cooperation^52^. This finding may be an evolutionary explanation of why unconditional cooperators emerge.

Second, there is another method that can resolve the dilemmas, this one using a norm that considers who has punished (the principle of justified punishment considering doers). We showed that this principle cannot completely solve the scoring dilemma and that another principle is required. By introducing a principle of generosity where evaluators assess cooperative behaviour against recipients with a bad reputation as good, these two principles can resolve the scoring dilemma and the dilemma of punishment simultaneously. Moreover, this method prevents the second-order free riders from invading, and thus, the additional social dilemma caused by the first method does not occur. This is why this method completely resolves the social dilemma as well as the first method. However, it has disadvantages in that it cannot work unless the efficiency of cooperation is high and the robustness of maintaining cooperation is lower than in the first method.

Comparing the two ways, we find that they have symmetrical aspects: While the first way considers the punishment from the side of the recipients, the second way considers the punishment from the side of the donors. The first way covers the case that the response of defection against bad persons should be to assign a good label or keep the label (condition 4 or 4′). This rule may reflect on our natural inclinations. For example, if we observe a defection against a bad person, we might think that there is a justifiable reason to defect because the recipient is bad. On the other hand, the second way covers the case that the response of defection against good persons should be to keep the label (condition 2′). This rule also may reflect our natural inclinations. If we observe a good person defecting against another good person, we might tend to think that there is a hidden reason for this behaviour, e.g., we may suppose that there was a bad event between the two persons though we don’t know for sure. However, if we see a bad person defecting against a good person, we might tend to think that this is as usual, and keep a bad impression of the doer. This deduction should be tested empirically in future work.

Our analysis has several limitations. We calculated replicator dynamics numerically, not analytically, and thus, we should conduct a more rigorous analysis in the future. We considered naive two strategies (ALLC and ALLD) only. Thus, it would be interesting to analyze a system among other competing strategies.

There are some theoretical studies on indirect reciprocity under private information; let us discuss our results in comparison with some of those. Although Okada et al. (2018)^49^ developed an analytical method to solve a system of private information without any approximation, they analysed only five rules. By comparison, we have exhaustively analysed all possible configurations in a specific assessment function space in a systematic way. Hilbe et al.^50^ analysed the original leading eight norms proposed by Ohtsuki and Iwasa^44^ by conducting individual-based simulations whose model is almost the same as ours. They show that some norms keep cooperative regimes while the majority do not.

To summarize our findings, behaviours with respect to recipients with good reputations should be rigorously evaluated, while behaviours of recipients with bad reputations should be generously assessed for maintaining stable cooperation. This insight may provide a basic assessment principle that applies to a wide range of subjects, from individual actions to national decisions.

## Methods

### Detailed model description

We consider a population consisting of an infinite set of well-mixed players with no interaction structure. Time is discrete in our model, and one game is played in every period. In the game, a pair of an actor and a recipient is randomly chosen from the population. The actor is given an option: cooperation (*C*) or defection (*D*). If the actor chooses *C*, the recipient gains a benefit *b* while the actor pays a cost *c* where *b* > *c* > *0* is assumed. If the actor chooses *D*, nothing happens. To generalize the model, we introduce an implementation error, in which there is a probability *e*_*1*_ of not cooperating when an actor intends to cooperate.

Our model deals with three types of player: perfect cooperators (*X*), perfect defectors (*Y*), and discriminators (*Z*). Both perfect cooperators and defectors behave unconditionally. The former always choose *C* and the latter always choose *D*. The discriminators privately give all players binary labels: either good (*G*) or bad (*B*). If a discriminator is chosen as a doer, the discriminator chooses *C* if and only if the recipient is labelled *G*.

We assume that each game is observed by only a finite number of discriminators. If a discriminator observes a game, it updates the label given to the actor of the game according to the reputation rule the discriminator adopts. In addition to the implementation error, we introduce a cognitive error in which there is a probability *e*_*2*_ that an assessing label is reversed when updating.

### Defining the assessment functions

To explore the conditions of the reputation rules for maintaining stable cooperation, we define assessment functions in the simplest cases. We set a private label *L* as binary: either good (*G*) or bad (*B*). In addition, we set an action (*A*) as binary: either cooperation (*C*) or defection (*D*). Thus, *L* = *{G,B}* and *A* = *{C,D}*. We then consider four types of method for updating the private labels (*U*): *U* = *{G,B,K,R}*, which represent, respectively, 'Assign *G*' , 'Assign *B*', 'Keep the label unchanged', and ‘Reverse the label between *G* and *B*’. The assessment function is defined as *f: L* × *A → U*.

Any rule is defined as four letters, while each letter is an element of the set *U*. The rules give patterns for updating the private labels of four situations. For example, *[GBKK]* is an assessment function. The first letter is a response to a situation in which a discriminator observes a game, the doer chooses *C* in the game, and the discriminator gives the *G* label to the recipient. The second letter is a response to a case in which the doer chooses *D* while the label of the recipient is *G*, the third letter is a response to a case in which the doer chooses *C* while the label of the recipient is *B*, and the fourth letter is a response to a case in which the doer chooses *D* while the label of the recipient is *D*.

The number of possible configurations of assessment functions is 256 because each letter has four options. For example, if a discriminator, say Alice, adopts an assessment function described as *[GBKK]*, any cooperator to a recipient whom Alice gives a good label is given (or kept) a good label, any defector to a recipient whom Alice gives a good label is given (or kept) a bad label, and the label of the actor (either good or bad) of any action (cooperation or defection) to a recipient whom Alice gives a bad label is not changed.

### Replicator equations of the population

We consider a continuous-entry model^33^: births and deaths occur randomly, and this changes the type distribution in the population. Let *x*, *y* and *z* be the fractions (population ratios) of *X*, *Y*, and *Z*, respectively, where *x* + *y* + *z* = *1*. To explore the evolutionary dynamics of the private labels, we assume that the time scale for natural selection is much slower than that of social interactions and updating the private labels^53^. Therefore, we can always assume that the frequency of labels is at its equilibrium value; that is, the expected probability that a player’s label is good has converged to a steady state. Let *g* be the fraction of players given good labels by *Z*. This fraction can be decomposed into *g*_*X*_, *g*_*Y*_ and *g*_*Z*_, where *g*_*s*_ is the fraction of players labelled good with type *s* in the set *S* = *{X,Y,Z}*. *g* = *x g*_*X*_ + *y g*_*Y*_ + *z g*_*Z*_. We consider a system of replicator equations. Let *P*_*s*_ be the expected payoff per cost of the game of an *s* type player where *s ∈ S*. The replicator dynamics are described as

$$ \dot{x} = x \, (P_{X} - \overline{P}) \, ,\dot{y} = y(P_{Y} - \overline{P}) \, , \, and\,\dot{z} = z \, (P_{Z} - \overline{P}) $$

where $\overline{P}$ = *x P*_*X*_ + *y P*_*Y*_ + *z P*_*Z*_ is the average payoff over the population. The expected payoffs of the three types are

$$ P_{X} = b\left( {x + zg_{X} } \right){-}c,P_{Y} = b(x + zg_{Y} ),{\text{ and}}\,P_{Z} = b\left( {x + zg_{Z} } \right) - cg $$

where we have omitted the factor *(1-e*_*1*_*)*.

## Dynamic analysis

We first solve for the values of *g* and *g*_*s*_ for *s ∈ S* given an assessment rule *f* and population distribution (*x*, *y*, *z*). Using the values of *g* and *g*_*s*_, the expected payoff of each type (*P*_*X*_, *P*_*Y*_, and *P*_*Z*_) is then calculated. Thus, the replicator dynamics show the dynamics of each population *(x,y,z)*. We need to check for the existence of cooperative stable points in the dynamics. Here, a cooperative stable point is one that satisfies two conditions. First, the point is either locally asymptotic or neutrally stable. Second, the average cooperation rate at that point (*x* + *zg*) exceeds a threshold point. A detailed analysis is given in the Supplementary Information.

## Supplementary information

Supplementary information
