# Supplementary material for: Two ways to overcome the three social dilemmas of indirect reciprocity
Source: Sci Rep. 2020 Oct 8;10:16799. doi: 10.1038/s41598-020-73564-5 (PMC7546724; doi:10.1038/s41598-020-73564-5)
Supplement: Supplementary file 1 — Supplementary information [file 41598_2020_73564_MOESM1_ESM.pdf]

Supplementary Information for Two ways to overcome the three social dilemmas of indirect reciprocity

Isamu Okada

August 2020

1 Numerical analysis of the replicator dynamics

Here, we analyze the replicator dynamics[1] defined in the Methods Section of the main text with respect to each assessment rule among the 256 types. For a given assessment rule denoted as $f = [f_1 f_2 f_3 f_4]$ defined in subsection 'Designing the assessment functions' in the Methods Section of the main text, we first solve for the values of g_s, where $s \in S = \{X, Y, Z\}$[2], are the types of player: perfect cooperator, perfect defector, and discriminator, respectively. To do so, we call f_i in $i \in \{1, 2, 3, 4\}$ a response of assessment rule f and any response is an element of $U = \{G, B, K, R\}$, i.e., the types of method for updating the private labels. Moreover, we use $H_u(s)$, where $s \in S$ and $u \in U$, as the expected response probability of G given to players who are in type s in accordance with the response u. If the response is G, the expected response is G despite its type s, and thus, $H_G(s) = 1$. If the response is B, the expected response is B, and thus, $H_B(s) = 0$. If the response is K, the expected response corresponds to the label before the game is played, and thus, $H_K(s) = g_s$. If the response is R, the expected response reverses the label before the game is played, and thus, $H_R(s) = 1 - g_s$.

The definitions of g_s in the case of f are presented in Eq.(1). First, we derive the definition of g_Y. The first term of the definition is the case that the recipient of a Y donor has a G label. In that case, the Y player chooses D, and thus, if a cognitive error does not occur (the probability is e_{21} where $e_{21} = 1 - e_2$), the probability the Y player's label is G is $H_{f2}(Y)$ because f_2 indicates a response to the case in which the doer chooses D, while the label of its recipient is G. If an error occurs, the probability the Y player's label is G is $(1 - H_{f2}(Y))$. The second term of the definition is the case that the recipient of the Y donor has a B label. In that case, we use f_4 instead of f_2 because f_4 indicates a response to the case in which the doer chooses D while the label of its recipient is B.

The definition of g_X is a little more complex than that of g_Y because we should consider the implementation error. The first term (describing that the recipient's label is G) is divided into four cases: the case with neither implementation error nor cognitive error, the case with no implementation error but a cognitive error, the case with an implementation error but no cognitive error, and the case with both an implementation error and a cognitive error. Here, we set $e_{11} = 1 - e_1$. In the first case, the calculation needs $H_{f1}(X)$ because f_1 indicates a response to the case in which the doer chooses C while the label of its recipient is G. In the final case, the calculation needs $(1 - H_{f2}(X))$. The second term, which considers the case of that the recipient's label is B, is also divided into four cases, as well as the first term. In this term, f_3 is used instead of f_1 in the first term because f_3 indicates a response to the case in which the doer chooses C while the label of its recipient is B.

Compared with g_X and g_Y, the definition of g_Z is even more complex because we must take care of the two images of the recipient in the eyes of the doer and in the eyes of the observer. To deal with it, we define a new parameter g_2 as the probability that any two Z players each give a player a G label. Using this variable, the probability that any two Z players give a different label to a player (one is G, while the other is B) is $g - g_2$, while the probability that any two Z players each give a player a B label is $1 - 2g + g_2$.

g is calculated as $xg_X + yg_Y + zg_Z$ and $g_2 = xg_{X2} + yg_{Y2} + zg_{Z2}$, where g_{s2} is the fraction of players labelled G by any two Z players with type $s \in S$. Because we assume that there is a finite number of observers among the infinite number of Z players in the game, $g_{s2} = (g_s)^2$ is satisfied. To explain this point, let us consider a player (say Alice) to whom any two Z players each give a G label. If the game is observed by one of the two Z players, the other player is never chosen as an observer because that probability goes to zero due to the finite number of observers. Therefore, the probability one of the two Z players labels Alice G is independent of whether the other Z player labels Alice G. This independence is derived from an idea on solitary observation by Okada et al.[13].

Therefore, the definitions of g_s in the case of f are

$$\begin{aligned}
g_X &= g[e_{11}e_{21}H_{f_1}(X) + e_{11}e_2(1 - H_{f_1}(X)) + e_1e_{21}H_{f_2}(X) + e_1e_2(1 - H_{f_2}(X))] \\
&+ (1-g)[e_{11}e_{21}H_{f_3}(X) + e_{11}e_2(1 - H_{f_3}(X)) + e_1e_{21}H_{f_4}(X) + e_1e_2(1 - H_{f_4}(X))] \\
g_Y &= g[e_{21}H_{f_2}(Y) + e_2(1 - H_{f_2}(Y))] + (1-g)[e_{21}H_{f_4}(Y) + e_2(1 - H_{f_4}(Y))] \\
g_Z &= g_2[e_{11}e_{21}H_{f_1}(Z) + e_{11}e_2(1 - H_{f_1}(Z)) + e_1e_{21}H_{f_2}(Z) + e_1e_2(1 - H_{f_2}(Z))] \\
&+ (g-g_2)[e_{11}e_{21}H_{f_3}(Z) + e_{11}e_2(1 - H_{f_3}(Z)) + e_1e_{21}H_{f_4}(Z) + e_1e_2(1 - H_{f_4}(Z))] \\
&+ (g-g_2)[e_{21}H_{f_2}(Z) + e_2(1 - H_{f_2}(Z))] + (1-2g+g_2)[e_{21}H_{f_4}(Z) + e_2(1 - H_{f_4}(Z))].
\end{aligned} \tag{1}$$

Therefore,

$$\begin{aligned}
g_X &= ge_{11}e_{22}H_{f_1}(X) + ge_1e_{22}H_{f_2}(X) + (1-g)e_{11}e_{22}H_{f_3}(X) + (1-g)e_1e_{22}H_{f_4}(X) + e_2 \\
g_Y &= ge_{22}H_{f_2}(Y) + (1-g)e_{22}H_{f_4}(Y) + e_2 \\
g_Z &= g_2e_{11}e_{22}H_{f_1}(Z) + e_{22}(g - g_2e_{11})H_{f_2}(Z) + (g-g_2)e_{11}e_{22}H_{f_3}(Z) \\
&+ e_{22}[1 - g(1+e_{11}) + g_2e_{11}]H_{f_4}(Z) + e_2
\end{aligned} \tag{2}$$

where $e_{22} = 1 - 2e_2$. Using this definition, we solve for the values of g_s given (x, y, z), because this system has five equations in five parameters: g_X, g_Y, g_Z, g, and g_2.

The values of g_s for $s \in S$ are

$$g_s = \sum_{i=1}^{4} J_{si}H_{f_i}(s) + e_2 \tag{3}$$

where

$$J = \begin{pmatrix} ge_{11}e_{22} & ge_1e_{22} & (1-g)e_{11}e_{22} & (1-g)e_1e_{22} \\ 0 & ge_{22} & 0 & (1-g)e_{22} \\ g_2e_{11}e_{22} & e_{22}(g - g_2e_{11}) & (g-g_2)e_{11}e_{22} & e_{22}[1 - g(1+e_{11}) + g_2e_{11}] \end{pmatrix}. \tag{4}$$

Therefore,

$$g_s = \frac{\sum_{f_i=R} J_{si} + \sum_{f_i=G} J_{si} + e_2}{1 - \sum_{f_i=K} J_{si} + \sum_{f_i=R} J_{si}}. \tag{5}$$

where $s \in \{X, Y\}$ if $e_2 > 0$.

Next, we describe an algorithm to numerically solve for the value of g. To do so, we consider solving the system of three equations in g_X, g_Y, and g_Z defined in Eq.(1). This system includes g and g_2, which are functions of the three variables (Note that $g = xg_X + yg_Y + zg_Z$ and $g_2 = x(g_X)^2 + y(g_Y)^2 + z(g_Z)^2$). Therefore, we must extend it to a system consisting of five variables and five equations.

For any given population distribution (x, y, z), both the defining equations of g_X and g_Y are functions of g, as shown in Eq.(5). Thus, given g, the values of g_X and g_Y are determined uniquely. Using the defining equation of g, g_Z is also determined uniquely. Using the defining equation of g_2, the value of g_2 is also determined uniquely. If the defining equation of g_Z is satisfied by substituting those values into it, they are a solution. We have confirmed that there is only one solution satisfying the above equation system in the range of $0 \leq g \leq 1$ for any of the parameter sets in our numerical calculations.

The algorithm is described as follows.

- Step 0. We set $g = 0$ and a new parameter $\epsilon = 10^{-3}$.

- Step 1. The values of g_X, g_Y, g_Z, and g_2 are solved for using the above method and the value of g. The values of those parameters are called g_X^*, g_Y^*, g_Z^*, and g_2^*, respectively
- Step 2. If the right-hand side of the defining function of g_Z is greater than g_Z^*, the value of g updates to $g+\epsilon$ and go back to Step 1. Otherwise, the algorithm stops.

Using the value of g determined above, the expected payoff of each type (P_X, P_Y, P_Z) can be determined for any given population distribution (x, y, z), and thus, the replicator dynamics can be solved numerically. We simulated the dynamics a specific parameter set for all 256 possible configurations of assessment rules. Fig. S1 shows the case of 16 assessment rules, denoted as $[GBf_3f_4]$ where $(f_3, f_4) \in U \times U$.

Now, let us explore cooperative stable points. There are inner fixed points which are neutrally stable in several cases. We check whether the cooperation rates $(x+zg)$ of each of those points exceed the threshold. If the value exceeds the threshold, the point is counted as a cooperative stable point. It is known that there are stable points in which the cooperation rates are high, there are also stable points in which the rates are quite low. The assessment rule $[GBBB]$, called shunning, is a well-known example of the latter case. Therefore, we need a threshold. It depends on the error rates and the cost-benefit ratio of the game (c/b). We chose the threshold values from experience, i.e., 80% when $b = 1.2$, 90% when $b = 1.5$, 92% when $b = 2$, 95% when $b = 3$, and 98% when $b = 5$. Note that we will only consider the case of $e_1 = e_2 = 1\%$ and $c = 1$.

As a result of an exhaustive exploration, we found that only the case $[GBKG]$ has such a point. For the parameter used to make Fig. S1, the point is $(x, y, z) = (21.44\%, 0.91\%, 77.65\%)$, and the average cooperation rate of that point is $x + zg = 92.34\%$.

Next, let us explore points that are asymptotically stable and for which the average cooperation rate exceeds the threshold. These points depend on the efficiency of cooperation, which is defined as b/c, i.e., the cost-benefit ratio of cooperation. If the efficiency of cooperation is 1.5, only 3 rules among all 256 rules have such a point: the assessment rule, population distribution, and average cooperation rate are, respectively, $\{[GBGG], (26.43\%, 0, 73.57\%), 95.81\%\}$, $\{[GBGK], (26.83\%, 0, 73.17\%), 95.83\%\}$, and $\{[GBKK], (30.32\%, 0, 69.68\%), 95.75\%\}$. Many studies have revealed that the image-scoring rule $[GBGB]$ has a continuum of equilibria on the boundary between the attractor on the line of $y = 0$ and the repeller on the line of $x = 0$. Thus, the population drifts along the continuum. If the efficiency of cooperation is 1.2, 2 rules ($[GBGG]$ and $[GBKG]$) lose their cooperative stable points. In summary, we have found a set of assessment rules which have a cooperative stable point, M_b, where b represents the efficiency of cooperation:

$$M_{1.2} = \{[GBuK] | u \in \{G, K\}\}, \quad (6)$$
$$M_{1.5} = \{[GBuv] | u, v \in \{G, K\}\}. \quad (7)$$

When the efficiency of cooperation equals 2.0 or more, the new rules join the group. As shown in Fig. S2, some rules of $[GKuv]$ have cooperative stable points at the top vertex of the triangle, which means that the point consists entirely of a DISC-type population. We find the following set:

$$M_5 = \{[GBuv], [GKuw] | u, v \in \{G, K\}, w \in U\}. \quad (8)$$

Finally, as the above analysis is for the case of $e_2 > 0$, we complement it with an analysis of the case of $e_2 = 0$. If an assessment f is any rule except for $[uKvK]$ where $(u, v) \in U \times U$, the denominator of Eq. (5) is strictly positive, and thus, the algorithm for finding a numerical solution on g can be applied. If an assessment f is denoted as $[uKvK]$ where $(u, v) \in U \times U$, g_Y is indefinite, and thus, the value of g can not be determined uniquely. Therefore, we will not deal with such rules. Thus, by analyzing the replicator dynamics, we have confirmed that the dynamics do not qualitatively change in the case of $e_2 > 0$.

2 Comparison with the results of previous studies on public assessment schemes

The vast number of studies on indirect reciprocity in public assessment schemes have revealed many features of the evolution of cooperation; here, we compare our results with theirs. First, we explore the cooperative stable points among all 256 assessment rules in the public assessment scheme. In this scheme, the defining equation of g_Z is replaced with the following equation, while the other definitions including those of g and P_s and the replicator dynamics remain the same.

$$g_Z^{Pub} = ge_{11}e_{22}H_{f_1}(Z) + ge_1e_{22}H_{f_2}(Z) + (1-g)e_{22}H_{f_4}(Z) + e_2 \quad (9)$$

Our numerical analysis of the replicator dynamics yielded a set of assessment rules that have a cooperative stable point when $b = 3$, $c = 1$, $e_1 = 1\%$, and $e_2 = 1\%$.

$$\begin{aligned} M_{1.2}^{Pub} &= \{[GBuv] | u \in U, v \in \{G, K\}\} \\ M_{1.5}^{Pub} &= \{[GBuv], [GBGR], [GBKB] | u \in U, v \in \{G, K\}\} \\ M_{5}^{Pub} &= \{[GBuv], [GBGR], [GBKB], [GBKR], [GKuv], [GRuv] | u \in U, v \in \{G, K\}\} \end{aligned} \quad (10)$$

It is interesting that $M_5 \not\subset M_5^{Pub}$ while $M_{1.2} \subset M_{1.2}^{Pub}$ and $M_{1.5} \subset M_{1.5}^{Pub}$. The assessment rules $\{[GKuw] | u, v \in \{G, K\}, w \in \{B, R\}\}$ have cooperative stable points in the private scheme, but do not have them in the public scheme. In this rule, the fixed points on the line ($y = 0$) in both schemes are a population in which ALLC and DISC coexist. At these points, ALLD cannot invade the population in the private scheme , but it can do so in the public scheme.

The assessment rules for a donor's label of the leading eight norms discovered by Ohtsuki and Iwasa in 2004[3] are described using 2 letters (G and B) in 8 cases consisting of all possible configurations of 2 labels (G and B) for the donor, 2 labels (G and B) for the recipient, and 2 actions (C and D), where G and B respectively represent Good and Bad, while C and D respectively represent cooperation and defection. Our notation uses 4 letters $U = \{G, B, K, R\}$ in 4 cases consisting of all possible configurations of 2 labels for the recipients and 2 actions. Table S1 compares the leading eight norms in our new notation.

Using the notation of Sigmund (2010)[4], $M_{1.5}$ is replaced as follows: $M_{1.5} = \{L1, L3, L4, L7\}$. Several studies on public assessment schemes consider these rules. Panchanathan and Boyd[5] analyzed L1 (say RDISC in their paper) and showed that this assessment rule dominates both ALLC and ALLD. Moreover, they analyzed CTFT, whose action rule is different while its assessment rule is the same as L1; they showed that this assessment rule dominates RDISC. This result is consistent with the exhaustive analysis performed by Ohtsuki and Iwasa[3]. This is why our L1 (RDISC[5]) is not included in the leading eight norms, while Ohtsuki and Iwasa's L1 (CTFT[5]) is included in the norms when not only assessment rules but also action rules are considered.

While less attention has been paid to L4[6, 7], many studies have analyzed L3[8, 9], and they have repeatedly shown that L3 performs well in public assessment schemes. In fact, thus some researchers call it a simple-standing rule. Our results for this rule are consistent with the previous studies, and we show that it also performs well in the private assessment scheme.

Panchanathan and Boyd(2011)[10] showed that L7 as well as L3 can invade a population consisting of ALLD. Since then, numerous studies hae analyzed the performance of L7. Sasaki et al. (2017)[11] called it a staying rule because it is equivalent to avoiding assessment when a recipient has bad reputation. Okada's studies[12, 13] showed that this rule performs well in the private assessment scheme.

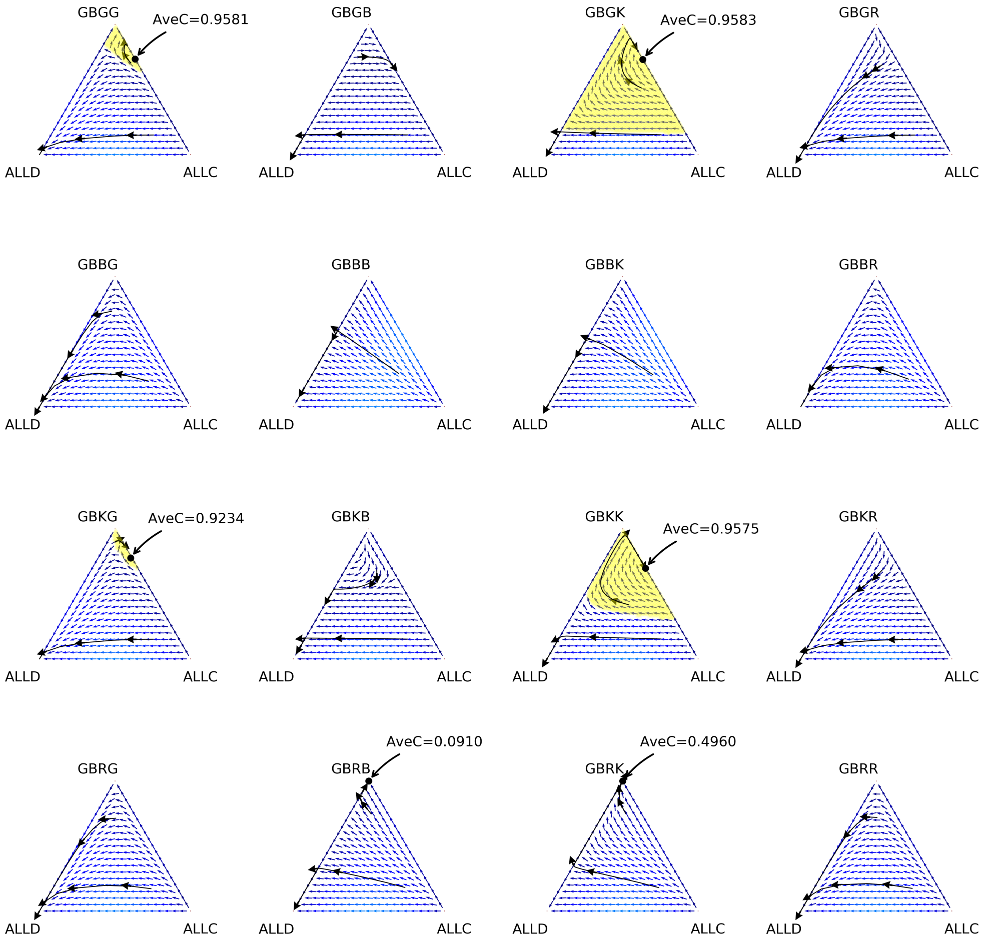

Fig. S1. The replicator dynamics of 16 assessment rules denoted as $[GBf_3f_4]$ where $(f_3, f_4) \in U \times U$. The triangles describe a simplex of the state space, $\{(x, y, z)|x + y + z = 1\}$, where x, y, and z are, respectively, non-negative real numbers denoting the frequencies of perfect cooperators (ALLC), perfect defectors (ALLD), and discriminators (DISC) who adopt the assessment rule. The arrows in the triangles show the direction of replicator dynamics at each point. Trajectories following the dynamics are also drawn. If there is a cooperative stable point, the average rate of cooperation (AveC) at that point is calculated and the basin of attraction is shown in yellow. Filled circles correspond to stable rest points. The parameter values are $b = 1.5$, $c = 1$, $e_1 = 1\%$, and $e_2 = 1\%$. This image is made by Python 3.

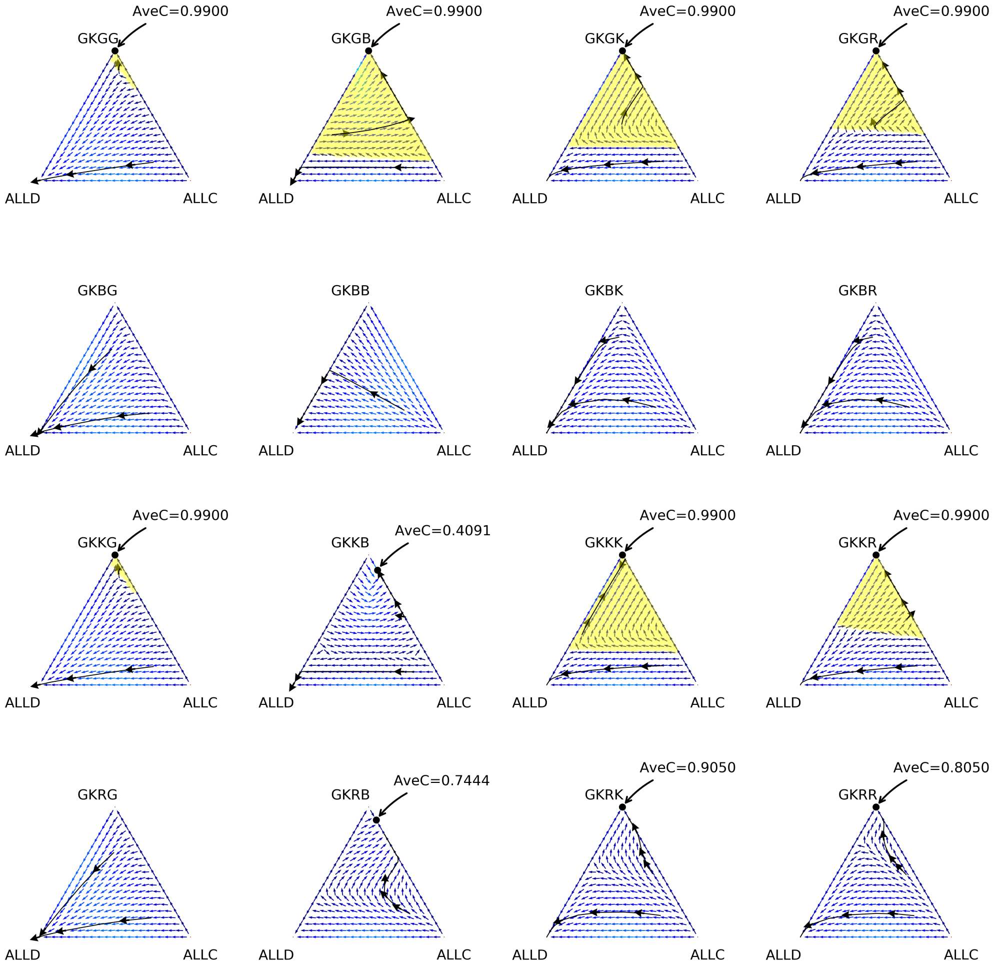

Fig. S2. The replicator dynamics of 16 assessment rules denoted as $[GKf_3f_4]$ where $(f_3, f_4) \in U \times U$. As well as Fig. S1, the triangles describe a simplex of the state space, $\{(x, y, z)|x + y + z = 1\}$. The parameter values are $b = 5$, $c = 1$, $e_1 = 1\%$, and $e_2 = 1\%$. This image is made by Python 3.

Table S1. New notation for the assessment rules of the leading eight norms

Norms	C to G	D to G	C to B	D to B
L1	G	B	G	K
L2	G	B	R	K
L3	G	B	G	G
L4	G	B	K	G
L5	G	B	R	G
L6	G	B	B	G
L7	G	B	K	K
L8	G	B	B	K

Note: We use the names of the norms (L1-L8) given by Sigmund(2010)[4]. The original leading eight norms proposed by Ohtsuki and Iwasa(2014)[3] define not only assessment rules but also action rules. In our analysis, each DISC player adopts a simple action rule (Sigmund (2010) calls it 'Co' in Table 4.2): cooperate with those whose label is G and defect against those whose label is B. Although the action rule of the original L3-L8 norms is 'Co', that of the original L1 and L2 is different from 'Co', which is called 'Self' by Sigmund(2010) in Table 4.2.

References

[1] Hofbauer, J. & Sigmund, K. Evolutionary games and population dynamics (Cambridge University Press, 1998)

[2] Brandt. H, & Sigmund, K. The good, the bad and the discriminator ? Errors in direct and indirect reciprocity. *J. Theor. Biol.* 239, 183-194 (2006)

[3] Ohtsuki, H. & Iwasa, Y. How should we define goodness? reputation dynamics in indirect reciprocity. *J. Theor. Biol.* 231, 107-120.(2004)

[4] Sigmund, K. The Calculus of Selfishness. (Princeton University Press, 2010)

[5] Panchanathan, K. & Boyd, R. A tale of two defectors: the importance of standing for evolution of indirect reciprocity. *J. Theor. Biol.* 224, 115-126 (2003)

[6] Ohtsuki, H. & Iwasa, Y. The leading eight: Social norms that can maintain cooperation by indirect reciprocity. *J. Theor. Biol.* 239, 435-444. (2006)

[7] Ohtsuki, H. & Iwasa, Y. Global analyses of evolutionary dynamics and exhaustive search for social norms that maintain cooperation by reputation. *J. Theor. Biol.* 244(3), 518-531 (2007)

[8] Sugden, R. The Economics of Rights, Cooperation and Welfare. (Oxford: Basil Blackwell, 1986)

[9] Milinski, M. et al. Cooperation through indirect reciprocity: image scoring or standing strategy? *Pr. R. Soc. B* 268. 2495-2501 (2001)

[10] Panchanathan, K. Two wrongs don't make a right: the initial viability of different assessment rules in the evolution of indirect reciprocity. *J. Theor. Biol.* 277, 48-54 (2011)

[11] Sasaki, T. et al. The evolution of conditional moral assessment in indirect reciprocity. *Sci. Rep.* 7, 41870 (2017)

[12] Okada, I. et al. Tolerant indirect reciprocity can boost social welfare through solidarity with unconditional cooperators in private monitoring. *Sci. Rep.* 7, 9737 (2017)

[13] Okada, I. et al. A solution for private assessment in indirect reciprocity using solitary observation. *J. Theor. Biol.* 455, 7-15 (2018)
